# A cross-sectional study on the mediating role of psychological capital in the relationship between workplace violence and professional commitment among nursing students

**DOI:** 10.3389/fpubh.2025.1629330

**Published:** 2025-09-25

**Authors:** Zhengrong Cai, Lili Jiao, JinJing Huang, Cui Peng, AiLing Chen, ZhouMin Shen, Yan Liu

**Affiliations:** ^1^Clinical Nursing Teaching and Research Section, The Second XiangYa Hospital of Central South University, Changsha, Hunan, China; ^2^Nursing Department, Hunan Provincial People’s Hospital, ChangSha, Hunan, China; ^3^School of Public Health, Hengyang Medical School, University of South China, Hengyang, Hunan, China

**Keywords:** professional commitment, psychological capital, workplace violence, nursing students, mediating role

## Abstract

**Background:**

Studies have documented associations among workplace violence (WPV), psychological capital (PsyCap), and professional commitment (PC). But limited research has investigated how PsyCap mediates the relationship between WPV and PC, particularly among nursing interns.

**Objective:**

This study assessed the current status of WPV, PsyCap, and PC among Chinese nursing students, analyzed thier interrelationships and further determined whether PsyCap mediated the association between WPV and PC.

**Methods:**

A cross-sectional survey of 520 nursing interns used validated instruments: a demographic questionnaire, the WPV Scale, the PC Scale, and the PsyCap Questionnaire (Chinese version of PCQ). Pearson correlation analyzed the relationships among PsyCap, WPV, and PC, while hierarchical regression tested the mediation model.

**Results:**

The total scores for PC and PsyCap among nursing students were (77.04 ± 17.04) and (91.90 ± 16.16), respectively. Approximately 31.54% of the participants reported experiencing WPV. PsyCap was inversely associated with WPV (*r* = −0.619, *p* = 0.024) and positively associated with PC (*r* = 0.620, *p* < 0.001), in contrast WPV showed a negative correlation with PC (*r* = −0.807, *p* = 0.005). Mediation analysis revealed that PsyCap mediated the WPV-PC relationship, accounting for 47.5% of the total effect.

**Conclusion:**

Nursing students exhibited moderate levels of PsyCap and PC, with the prevalence of WPV being slightly lower than reported in comparable studies. And PsyCap fully mediated the relationship between WPV and PC. These findings revealed that nursing administrators and educators should enhance and develop nursing students’ PsyCap to lower the adverse effects of WPV and promote higher levels of PC.

## 1 Introduction

The growing global population, coupled with the rapid aging of societies, has placed unprecedented pressure on healthcare systems worldwide, exacerbating the demand for qualified nursing professionals (1). Recent projections from *the State of the World’s Nursing report 2020* indicate a looming deficit of approximately 5.7 million nurses by 2030, with China facing an even more severe shortage (2). This scarcity is attributed to insufficient professional training capacity in higher education institutions and high turnover rates among practicing nurses. Among the various factors contributing to nurse attrition, professional commitment (PC) is recognized as a critical influencing and moderating variable, not only mitigating turnover intentions but also enhancing job satisfaction, thereby contributing to the health, stability, and sustainable growth of nursing professionals (3, 4). PC encompasses an individual’s dedication to their chosen career, characterized by a strong sense of nurses’ identification, loyalty, and active engagement in professional practice (5). Nursing interns, who represent the next generation of healthcare professionals, undergo a crucial period during clinical training where their PC is shaped. The level of PC formed during this stage is significantly correlated with their future commitment (6), potentially mitigating the nursing shortage. Although PC profoundly influences nursing students’ career decisions (7, 8), directly affecting their retention intentions and career choices, studies indicate that the PC of Chinese nursing students is mostly still at a moderate level (6, 9). This is particularly concerning given that clinical internships mark a critical transition from the structured academic setting to the dynamic and often high-pressure clinical environment. And this phase is instrumental in fostering the competencies and mindset required for a sustainable nursing career. Consequently, the PC of nursing students has garnered increasing attention from educators and researchers.

Clinical internship is a pivotal part of nursing education, shaping professional attitudes, emotional engagement, and occupational identity. The work environment plays a decisive role in this process. And workplace violence (WPV), a prevalent negative occurrence in nursing settings, has become increasingly common. A systematic review (10) reported that 61.9% of healthcare workers worldwide have encountered various forms of WPV, with non-physical aggression (42.5%) being more prevalent than physical violence (24.4%) in the past year. Within non-physical violence, verbal violence is the most frequent reported, followed by threats and sexual harassment. In China, escalating medical disputes over the past two decades have heightened occupational risks for healthcare professionals (11, 12). A meta-analysis (13) revealed that 65.4% of Chinese healthcare workers experienced WPV with a rate surpassing global averages, and physical violence (13.7%), psychological violence (50.8%), verbal abuse (61.2%), threats (39.4%), and sexual harassment (6.3%) also were distressingly common. Psychiatric nursing staff were found to be at greatest risk, with a staggering 78% prevalence rate. Compared to other professions, nursing is among the most susceptible to WPV, reflecting a severe and widespread global issue (14, 15). And over 40% of psychiatric nurses in Japan reported WPV within a single year (16). In Australia, 73.7% of emergency department nurses reported experiencing WPV over the past 2 years (17). Similarly, a study conducted in Latin America found that 59.2% of healthcare workers had been exposed to WPV (18). Consistent with these findings, a cross-sectional survey in China revealed that 49.12% of nurses experienced WPV within the previous 6 months (19). Notably, nursing interns, who are still developing their clinical skills, are especially susceptible to various forms of WPV during clinical training (20–22).

The consequences of WPV for healthcare workers include adverse psychological states, reduced job satisfaction, increased turnover intentions, and diminished quality of life (23, 24). Multiple studies demonstrate that WPV significantly impairs mental health, even leading to severe psychiatric conditions such as depression (25, 26). These negative outcomes exacerbate turnover intentions and deteriorate doctor–patient relationships (27). Long-term exposure to such environments may result in substantial losses of healthcare personnel and a decline in service quality (28). A qualitative study found that nursing students who experienced harassment reported diminished care quality for patients (29). Those subjected to verbal abuse exhibited higher turnover intentions than their unaffected peers (30). Recently, attention has shifted toward examining the impact of WPV on PC, particularly among nursing students, with findings suggesting that WPV exposure undermines their dedication to the field (22, 31). Despite these insights, the precise pathways through which WPV influences PC remain poorly understood, warranting further investigation.

Psychological capital (PsyCap), a key concept in positive organizational behavior, refers to an individual’s positive developmental state characterized by four key dimensions: self-efficacy (confidence in one’s abilities), hope (perseverance toward goals), resilience (capacity to recover from adversity), and optimism (positive attribution of future outcomes) (32). PsyCap has been identified as a crucial resource in mitigating stress and reducing turnover (33). For instance, a study involving Chinese university faculty, including medical professionals, confirmed that PsyCap acts as a psychological safeguard against depression, mediating the adverse effects of occupational stress on mental health (34). And various studies have linked PsyCap to PC and WPV. For example, a study of 249 Chinese clinical medical undergraduates demonstrated that PsyCap positively influences PC (35). Another study of 710 Chinese nursing students revealed that self-efficacy, a dimension of PsyCap, significantly positively correlated with and predicted PC through the mediation of life meaning (9). Additionally, self-efficacy was negatively correlated with WPV and found to alleviate occupational stress and enhance job satisfaction among Chinese physicians exposed to WPV (36). Additional evidence suggests that PsyCap mediates the impact of WPV on professional identity in Chinese psychiatric nurses (37). However, to our knowledge, no prior study has specifically investigated whether PsyCap mediates the relationship between WPV and PC among Chinese nursing students. Given the existing literature, we propose that PsyCap serves as a mediating mechanism linking WPV to PC in this population.

**Figure 1:**
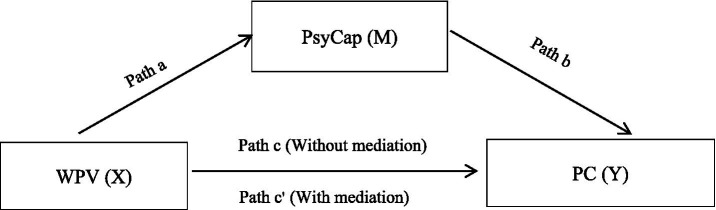
The hypothetical model of the relationship between WPV and PC mediated by PsyCap. WPV, workplace violence; PsyCap, psychological capital; PC, professional commitment.

This study investigates the current status of WPV, PsyCap, and PC among Chinese nursing students, exploring their interrelationships and the mediating effect of PsyCap. We hypothesize that: WPV is negatively correlated with PC, and PsyCap mediates the relationship between WPV and PC in nursing interns (Figure 1).

## 2 Method

### 2.1 Study design

A cross-sectional survey utilizing convenience sampling was conducted from January to March 2025 at four tertiary Grade A general hospitals in Hunan Province, China: the Xiangya Second Hospital of Central South University, Hunan Provincial People’s Hospital, the First Affiliated Hospital of University of South China, and the First Affiliated Hospital of Hunan University of Medicine. The inclusion criteria were: nursing interns with ≥6 months of continuous clinical practice, including diploma and undergraduate students, who voluntarily participated. And the exclusion criteria were: interns in non-clinical positions (e.g., nursing administration, supply room) or those who discontinued their internship during the survey period.

### 2.2 Data collection

The sample size was calculated using the formula for cross-sectional studies: *N* = *Z*_*α*/2_^2^ × *p*(1 − *p*)/*d*^2^, where *Z*_α/2_ is the *Z*-value corresponding to the chosen α level (*α* = 0.05, *Z*_α/2_ = 1.96), p is the estimated prevalence rate of WPV [reported as approximately 49% in the literature (19)], and d is the margin of error (set at 5%). The minimum sample size was calculated to be 384. Considering a potential 20% rate of invalid responses, a total of 460 participants were targeted. In this study, questionnaires were distributed online via Questionnaire Star, and a total of 545 responses were collected. After excluding those with patterned or unreliable answers, 520 valid questionnaires were retained, resulting in a valid response rate of 95.41%.

### 2.3 Ethical approval

This study was conducted in accordance with the Declaration of Helsinki and was approved by the Clinical Research Ethics Committee of the Second Xiangya Hospital, Central South University (Ethics Approval Number: LYF20250127). Written informed consent was obtained from all participants, who were informed of their right to withdraw at any time without justification or career repercussions. Data were collected anonymously and maintained with strict confidentiality for research purposes only.

### 2.4 Measurement instruments

#### 2.4.1 Demographic characteristics questionnaire

Study-specific demographic data were gathered using a customized survey instrument that recorded gender (male/female), education level (Junior college/undergraduate), single-child status, parent–child relationship (harmonious/moderate/discordant/poor), prior nurse–patient communication training, voluntary choice of nursing major, and career affinity.

#### 2.4.2 PsyCap measurement

The PsyCap Questionnaire (PCQ), originally developed by Luthans et al. (38) and later adapted into Chinese version by Luo and He (39), included 20 items assessing four key psychological constructs: self-efficacy (6 items), hope (6 items), resilience (5 items), and optimism (3 items). Participants rated their responses on a 6-point Likert scale, with options ranging from 1 (strongly disagree) to 6 (strongly agree), yielding a total possible score ranging from 20 to 120. Based on established cutoffs, scores were categorized as low (<48), moderate (48–105), or high (>105). The Cronbach’s *α* coefficients for total PCQ and subscales ranged 0.718–0.923, and test–retest reliability was 0.774–0.874.

#### 2.4.3 WPV measurement

To evaluate WPV incidents occurring within the preceding six-month period, this study employed the WPV Scale, an instrument originally developed by Chen (40). This validated tool assesses four primary types of violence: verbal abuse, verbal threats, physical assaults, and sexual harassment. In addition to measuring the frequency and severity of these events, the scale captures contextual details, including perpetrator profiles and contributing factors underlying violent encounters. The scale demonstrated excellent reliability (Cronbach’s *α* = 0.986) and validity (KMO = 0.948; Bartlett’s test = 109,238.644, *p* < 0.001).

#### 2.4.4 PC measurement

The 23-item PC Scale developed by Lu et al. (41) assessed three dimensions: willingness to strive (9 items), intent to remain in the profession (8 items), and professional value recognition (6 items). Responses were recorded on a 5-point Likert scale ranging from 1 (very uncertain) to 5 (very certain), with total scores ranging from 23 to 115 (higher scores indicating greater commitment). The scale showed good reliability (Cronbach’s α = 0.93; test–retest reliability = 0.81).

### 2.5 Statistical analysis

Data were analyzed using SPSS 27.0 and R 4.2. Continuous variables that followed a normal distribution were summarized using means and standard deviations (mean ± SD), whereas categorical variables were expressed as absolute frequencies and relative percentages. Analysis of variance (ANOVA) was used to compare differences in PsyCap and PC scores across different groups of nursing students. Chi-square tests were employed to analyze differences in WPV incidence rates. Bivariate associations between PsyCap, WPV, and PC were examined using Pearson correlation. Furthermore, to explore the potential mediating role of PsyCap in the relationship between WPV and PC, a hierarchical multiple regression analysis was performed.

## 3 Result

### 3.1 General characteristics and statistical description

A total of 545 questionnaires were collected in this study, with 520 valid questionnaires screened, resulting in an effective response rate of 95.41%. Among the respondents, 63 were male (12.11%) and 457 were female (87.89%). A total of 164 nursing students reported experiencing WPV, accounting for 31.54% of the participants. The proportion of female students experiencing WPV (31.73%) was slightly higher than that of male students (30.15%). Verbal violence was the most prevalent form, with an incidence rate of 90.24% (148/164), followed by physical violence at 14.63% (24/164) and sexual violence at 19.51% (32/164). The mean scores for PsyCap and PC were 91.90 ± 16.16 and 77.04 ± 17.05, respectively, with male participants scoring higher than females in both measures. Gender and single-child status showed no significant influence on WPV, PsyCap, or PC (*p* > 0.05). Participants who voluntarily chose nursing as their major demonstrated markedly higher PsyCap (*p* = 0.016) and PC (*p* < 0.001) scores than those who did not. Those who expressed a strong interest in the nursing profession experienced less WPV (*p* = 0.007) and exhibited higher PsyCap and PC scores compared to their less interested counterparts (*p* < 0.001). Educational background significantly influenced WPV, PsyCap, and PC (*p* < 0.001). Nursing students who had participated in nurse–patient communication training scored higher in PsyCap (*p* = 0.013) and PC (*p* = 0.048), though no statistically significant effect was observed on WPV exposure. Additionally, academically high-achieving students showed elevated PsyCap (*p* = 0.003) and PC scores (*p* < 0.001) (Table 1).

**Table 1:** WPV, PsyCap, and PC across different characteristics.

Items	Group	WPV	PsyCap	PC
Gender	Male	19(63)	93.06 ± 18.48	78.59 ± 18.96
	Female	145(457)	91.74 ± 15.83	76.83 ± 16.78
*F*/*X*^2^		0.063	0.077	0.639
*P*		0.802	0.782	0.424
Education	Junior College	102(267)	89.28 ± 15.68	73.81 ± 16.47
	Undergraduate	62(253)	94.66 ± 16.23	80.45 ± 17.01
*F*/*X*^2^		44.788	0.067	2.707
*P*		<0.001	<0.001	<0.001
Single-child status	Yes	26(79)	93.57 ± 18.89	77.37 ± 19.13
	No	138(441)	91.6 ± 15.63	76.99 ± 16.67
*F*/*X*^2^		0.081	1.476	0.037
*P*		0.776	0.224	0.847
Parent-child relationship	Harmonious	126(429)	96.86 ± 16.51	77.96 ± 17.49
	Moderate	36(84)	87.63 ± 16.04	72.98 ± 13.98
	Discordant	2(6)	85.33 ± 6.12	69.83 ± 17.82
	Poor	0(1)	64	69
*F*/*X*^2^		6.356	14.465	12.935
*P*		0.58	<0.001	0.002
Voluntary choice of nursing major	Voluntary choice	69(250)	94.91 ± 18.09	83.20 ± 17.53
	Parental/Others’ influence	37(122)	89.88 ± 13.83	72.53 ± 14.14
	Employment pressure	23(57)	87.84 ± 14.01	69.21 ± 11.76
	Program adjustment	35(90)	88.82 ± 12.97	70.96 ± 16.18
*F*/*X*^2^		6.290	10.275	63.383
*P*		0.98	0.016	<0.01
Career affinity	Yes	66(167)	95.30 ± 15.94	83.16 ± 15.62
	No	98(353)	84.71 ± 14.18	64.13 ± 11.99
*F*/*X*^2^		7.260	31.860	108.516
*P*		0.007	<0.001	<0.001
Prior nurse-patient communication	Yes	90(293)	93.95 ± 16.54	77.98 ± 17.72
	No	74(227)	89.26 ± 15.29	75.83 ± 16.09
*F*/*X*^2^		0.210	6.130	3.911
*P*		0.647	0.013	0.048
Final academic performance	Excellent	83(270)	94.14 ± 17.50	79.86 ± 17.48
	Good	78(233)	89.90 ± 14.02	74.12 ± 15.81
	Pass	2(16)	84.44 ± 16.48	73.38 ± 19.84
	Fail	1(1)	74	59
*F*/*X*^2^		5.007	12.356	20.248
*P*		0.123	0.003	<0.001

### 3.2 Relationships among nursing students’ PsyCap, WPV, and PC

Pearson correlation analysis revealed significant associations among the study variables: WPV was negatively correlated with PsyCap (*r* = −0.619, *p* = 0.024) and PC (*r* = −0.807, *p* = 0.005). In contrast, a strong positive correlation was observed between PsyCap and PC (*r* = 0.620, *p* < 0.001).

### 3.3 Mediating role of PsyCap between WPV and PC

**Figure 2:**
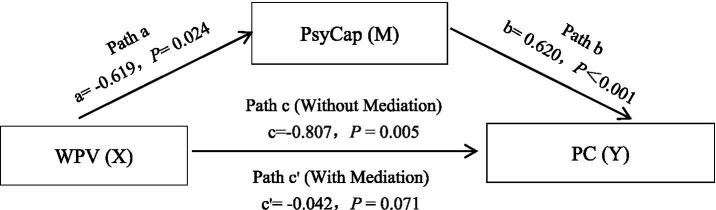
The mediating pathway of PsyCap in the relationship between WPV and PC.

Figure 2 demonstrates the mediating role of PsyCap in linking WPV and PC. As presented in Table 2, WPV exerted a negative effect on PC (*c* = −0.807, *t* = −2.804, *p* = 0.005). WPV negatively influenced PsyCap (*a* = −0.619, *t* = −2.262, *p* = 0.024), while PsyCap showed a strong positive correlation with PC (*b* = 0.620, *t* = 16.568, *p* < 0.001). No direct effect was observed between WPV and PC (*c*’ = −0.424, *t* = −1.811, *p* = 0.071), indicating that PsyCap fully mediated the relationship between WPV and PC.

**Table 2:** The mediating role of PsyCap between WPV and PC.

Effect type	Path relationship	Coefficient (*B*)	*t*	*P*	*R* ^2^	*F*
Total effect (c)	X → Y	−0.807	−2.804	0.005	0.015	7.864
Mediating effect (a)	X → M	−0.619	−2.262	0.024	0.010	5.118
Mediating effect (b)	M → Y	0.620	16.568	<0.001	0.354	143.266
Direct effect (c’)	X → Y (control M)	−0.424	−1.811	0.071		

The calculated mediation effect, derived from the product of coefficients (a × b, −0.619 × 0.620 ≈ −0.383). This represents 47.5% of the total observed effect (0.383/0.807), demonstrating that nearly half of WPV’s influence on PC operates through the PsyCap pathway.

## 4 Discussion

### 4.1 Current status of WPV, PC and PsyCap among nursing students

According to a recent investigation (42), which involved 526 nursing students across six institutions in China’s Hubei, Guangdong, and Gansu provinces, approximately 58.17% of respondents encountered WPV during clinical internships, predominantly in the form of verbal abuse. In our study, 31.54% of nursing students experienced WPV, slightly lower than current reports, with verbal violence also being the most frequent. This discrepancy may be explained by regional cultural distinctions and variations in hospital levels surveyed. Factors such as gender, single-child status, voluntary choice of nursing as a major, prior communication training, and academic performance showed no significant correlation with WPV frequency. However, students who expressed passion for the nursing profession experienced less WPV, and undergraduates reported fewer incidents than junior college students.

Research indicates that nursing students frequently encountering WPV face significant detrimental effects on their professional identity, clinical training motivation, and performance quality (30). Such exposure not only compromises patient safety but also poses long-term risks to the sustainability of the nursing workforce. The consequences of bullying during clinical placements manifest across multiple dimensions. On an individual level, affected students commonly exhibit psychological distress, including anxiety, depressive symptoms, impaired self-regulation, and in severe cases, post-traumatic stress reactions (43). Organizationally, WPV contributes to decreased staff engagement, higher attrition rates, and increased absenteeism (44), all of which compromise patient care quality and safety. Professionally, these experiences profoundly affect students’ career development and aspirations, undermining their professional identity and confidence, and even prompting some students to reconsider their future in nursing (45).

Nursing administrators should establish comprehensive WPV prevention and response mechanisms. This includes implementing clear zero-tolerance policies, standardizing reporting procedures for violent incidents, and ensuring timely support and protection for students who experience violence. Additionally, healthcare institutions should enhance educational training for nursing students to develop competencies in handling violence situations, such as offering courses in communication skills, conflict resolution, and emotional management. Nursing educators should integrate WPV prevention and response into the nursing curriculum, using case studies and role-playing to prepare students before internships. And educators should encourage nursing students to actively participate in hospital-based violence prevention initiatives, fostering their social responsibility and professional dedication.

This study indicated that nursing students exhibited a moderate degree of professional commitment, consistent with existing research in the field (9). Specifically, students who voluntarily chose nursing, expressed passion for the profession, had prior patient–nurse communication training, and excelled academically exhibited higher professional commitment. PC in nursing is a continuous and dynamic process that begins during university education (46) and may directly influence commitment levels after becoming registered nurses. Therefore, nursing schools should strengthen systematic, evidence-based professional education to help students establish proper career values. Additionally, offering career planning courses can clarify professional goals, and inviting experienced nurses to share insights can inspire passion for the field.

Contemporary research has identified nursing as an occupational field with elevated risks for mental health disturbances. PsyCap, particularly among nurses, is increasingly valuable for improving mental well-being. Studies suggest that PsyCap constitutes an essential factor in helping nurses sustain mental equilibrium amidst professional pressures (47). Another systematic research (48) revealed that Asian nurses had the lowest PsyCap scores, possibly due to population growth, rising demand for nursing services and workforce shortages. Current findings indicate that Chinese nursing students exhibit moderate levels of PsyCap (49), consistent with this study. Students with harmonious family relationships, voluntary career choice, passion for nursing, and prior communication training demonstrated higher PsyCap, suggesting that PC influences PsyCap. Nursing educators should prioritize mental health interventions and provide training programs to enhance PsyCap development.

### 4.2 Relationship between WPV, PC and PsyCap

Our results demonstrate an inverse correlation between WPV and PC, supporting findings from a qualitative study of 12 nursing students (50) and aligning with a large-scale cross-sectional research conducted across multiple centers in China (22). Among students exposed to violence, PC was negatively affected, significantly reducing confidence and motivation in nursing, potentially influencing career choices and future turnover rates. Hospital administrators should establish comprehensive management mechanisms for WPV prevention, response, reporting, and handing to effectively reduce WPV incidence, improve coping abilities, and enhance professional commitment.

Research indicates that PsyCap is positively correlated with PC among Chinese undergraduate medical students (22), a finding replicated in this study. This indicates that PsyCap serves as a beneficial resource for improving PC. Nursing educators can enhance students’ psychological resilience through PsyCap training, thereby strengthening commitment, increasing career loyalty, and reducing turnover. Additionally, this finding suggests an inverse relationship between PsyCap and WPV, aligning with prior research (26). Given that PsyCap acts as a favorable factor in mitigating mental health issues such as depressive symptoms (51), it may mitigate the adverse effects of WPV on healthcare workers.

### 4.3 The mediating role of PsyCap in the relationship between WPV and PC

Our research identified PsyCap as a mediator between WPV and PC among Chinese nursing students, meaning it serves as a protective factor that helps maintain PC despite WPV. Yao et al. (47) also demonstrate that PsyCap can modify the impact of WPV on stress and job satisfaction. A possible explanation is that students with higher PsyCap may counteract WPV’s effects through increased effort and adaptive coping strategies, preserving their commitment. In recent years, studies from low- and middle-income countries and conflict-affected regions have also revealed the significant impact of WPV on the occupational commitment of nursing staff. For example, a study on nurses in Pakistan found that WPV was significantly associated with burnout and turnover intention, with PsyCap playing a moderating role (52). In Angola, a study focusing on interns in conflict areas indicated that psychological resilience training significantly improved job satisfaction (53). These findings further support the potential role of psychological capital in mitigating the negative effects of WPV.

This study is the first to validate the full mediating role of PsyCap in the relationship between WPV and PC among nursing interns in China, addressing a gap in the existing literature. Given the growing global shortage of nursing human resources, the findings of this study have important implications for international nursing education. On one hand, nursing educators should integrate PsyCap interventions into the curriculum to enhance students’ psychological resilience in coping with WPV. On the other hand, policymakers should consider PsyCap training as a key strategy to prevent nurse turnover and improve the stability of the nursing workforce. Therefore, this study not only provides empirical evidence for nursing education reform in China but also offers a theoretical framework and practical pathway for global nursing human resource management. Last but not least, given that PsyCap is malleable and manageable, hospital administrators should implement PsyCap interventions, such as mental health education, lectures, group counseling, and professional training, to enhance students’ resilience against WPV.

Research demonstrates that effective measures can improve PsyCap. Currently, the PsyCap Intervention (PCI) model has been developed (54) and tested among Chinese employees (55), proving effectiveness in enhancing psychological resilience, though limited to online self-learning. Therefore, universities and hospitals should develop tailored PsyCap interventions for nursing students. For example, universities could integrate PsyCap training into pre-clinical internship courses, while hospitals could establish a “zero-tolerance” policy, standardize incident reporting procedures, and set up psychological support hotlines for nursing students. Strengthening PsyCap can not only mitigate the negative impact of WPV on PC but also promote personal growth, career development, job satisfaction, and teaching quality, ultimately sustaining or improving PC levels.

### 4.4 Strengths and limitations

This study verified the mediating effect of PsyCap in the association between WPV and PC among nursing students, which has not been previously reported. However, several limitations must be acknowledged. First, the reliance on self-reported measures for data collection may lead to potential biases, such as memory inaccuracies or subjective reporting. Second, the participant pool was limited to tertiary Grade A hospitals in Hunan Province, omitting healthcare institutions of varying tiers or geographical locations. This restricted sampling may affect the broader applicability of the findings, warranting future research with more diverse hospital settings and regional representation. Third, the cross-sectional nature of the study design hinders the ability to infer causality among the variables. Longitudinal studies with multiple time points are recommended to better assess temporal dynamics and establish causal pathways linking WPV, PsyCap, and PC. Fourth, this study did not control for potential confounding variables that may influence the observed relationships, such as prior trauma, mental health status, and institutional culture. These factors may potentially affect the associations among WPV, PsyCap, and PC. Future studies should aim to control for these variables. Finally, this study focused solely on PsyCap as a mediator, potentially overlooking other underlying mediating variables. For instance, factors such as nursing managers’ support, work engagement, and emotional states may also mediate the relationship between WPV and PC. Future studies should broaden the scope of potential mediators to explore additional underlying mechanisms, thereby providing a more comprehensive understanding of the pathways through which WPV influences PC.

## 5 Conclusion

This study investigated the current status of PsyCap, WPV and PC among nursing students, examined the relationships among these three factors, and further explored the mediating role of PsyCap between WPV and PC among Chinese nursing students, providing valuable insights for nursing administrators and educators. First, nursing administrators and educators should attach great importance to the issue of WPV by implementing effective measures to prevent and reduce its occurrence while enhancing nursing students’ coping capacities. Second, given the significant direct negative impact of WPV on nursing students’ PC, nursing educators should establish comprehensive management systems, strengthen student training and communication, and foster a safe, harmonious, and violence-free work environment. Additionally, considering that PsyCap mediates the link between WPV and PC, nursing leaders and educators should prioritize enhancing and developing nursing students’ psychological resources. This approach can help counteract the adverse impacts of WPV and support the long-term growth of the nursing field.

## Data Availability

The original contributions presented in the study are included in the article/supplementary material, further inquiries can be directed to the corresponding author.
